# REPdenovo: Inferring *De Novo* Repeat Motifs from Short Sequence Reads

**DOI:** 10.1371/journal.pone.0150719

**Published:** 2016-03-15

**Authors:** Chong Chu, Rasmus Nielsen, Yufeng Wu

**Affiliations:** 1 Department of Computer Science and Engineering, University of Connecticut, Storrs, CT 06269, United States of America; 2 Department of Integrative Biology, University of California, Berkeley, CA 94720, United States of America; CNRS UMR7622 & University Paris 6 Pierre-et-Marie-Curie, FRANCE

## Abstract

Repeat elements are important components of eukaryotic genomes. One limitation in our understanding of repeat elements is that most analyses rely on reference genomes that are incomplete and often contain missing data in highly repetitive regions that are difficult to assemble. To overcome this problem we develop a new method, REPdenovo, which assembles repeat sequences directly from raw shotgun sequencing data. REPdenovo can construct various types of repeats that are highly repetitive and have low sequence divergence within copies. We show that REPdenovo is substantially better than existing methods both in terms of the number and the completeness of the repeat sequences that it recovers. The key advantage of REPdenovo is that it can reconstruct long repeats from sequence reads. We apply the method to human data and discover a number of potentially new repeats sequences that have been missed by previous repeat annotations. Many of these sequences are incorporated into various parasite genomes, possibly because the filtering process for host DNA involved in the sequencing of the parasite genomes failed to exclude the host derived repeat sequences. REPdenovo is a new powerful computational tool for annotating genomes and for addressing questions regarding the evolution of repeat families. The software tool, REPdenovo, is available for download at https://github.com/Reedwarbler/REPdenovo.

## Introduction

Most genomes, and in particular mammalian genomes, consist of large amounts of repeat elements. A repeat is a segment of DNA that appears multiple times in the genome in identical or near-identical form. In this paper, we use “repeat” to refer to a consensus of all the copies of the same repeat element. There are many types of repeats [1–3]. Transposable elements (TEs) are perhaps the most well-known. They are believed to constitute 25% to 40% of most mammalian genomes [2–5] and can amplify themselves in the genome using various mechanisms, typically involving RNA intermediates. In humans the most common TEs are Long Interspersed Elements (LINE-1s or L1s), Short Interspersed Element (SINEs), and Long Terminal Repeats (LTRs), comprising approx. 17%, 11% and 8% of the human genome, respectively. Other common repeat elements include various types of satellites.

Identifying repeats in a genome is a long-standing research problem. There are many computational approaches and software tools for the analysis of repeat composition [6–10]. One type of repeat analysis tools rely on curated repeat libraries to identify repeats. The most popular of these is RepeatMasker [6], which aligns genomic sequences to known consensus repeat sequences given by libraries such as Repbase [11] and Dfam [12]. While RepeatMasker has been used extensively in the literature and led to many interesting discoveries, a limitation is that it needs a library of known repeat consensus sequences. Such repeat sequences are usually not available for many newly sequenced organisms. Alternatively, many existing methods [7–10] identify repeats by analysis of reference genomes. However, many genomes are poorly assembled, particularly in regions of high repeat content. Therefore, most existing methods can not find novel repeats that are not present in the curated library of repeats or in a reference genome. For organisms with little repeat annotation and without a good reference genome, there are few tools available for characterizing repeat content. Even for organisms such as humans with good reference genomes, there are often missing bases in regions of high repeat content. The human genome may therefore still harbor uncharacterized repeat elements.

In principle, finding repeats directly from sequence data may be appropriate for situations where there is no good reference genome or we want to find repeats that are not present in the reference genome. Recently, methods that analyze repeats based on sequence data start to appear. One such method is RepARK [13]. RepARK can assemble repeats directly from sequence reads without reference genomes. However, experiments on RepARK show that there is still great room to improve the repeat assembly.

In this paper, we present a new approach for *de novo* assembly of repeat elements, called REPdenovo. Similar to RepARK, REPdenovo constructs repeats from sequence reads directly and does not need a reference genome. REPdenovo aims at constructing repeats that have relatively high copy numbers and low sequence divergence within copies of the repeats. The repeats can be of various types, e.g. TE or satellites. The main advantage of REPdenovo is that it implements more accurate repeat assembly algorithms than RepARK. Using real data, we demonstrate that REPdenovo outperforms RepARK in terms of completeness and number of long repeats constructed. We also analyze sequence data from humans, and report potentially new human repeat elements missed by previous analyses. We also provide supporting evidence which shows many of these repeats are likely to be real.

## Background

There are several existing computational approaches for finding TEs from short sequence reads [13, 14]. The method in [14] assumes a reference genome is available, and finds repeats from sequence reads using the reference. A major drawback is that there is no high-quality reference genomes for many organisms. In principle, one can use short reads to assemble a reference genome. However, repetitive regions are usually more difficult to assemble. This leads to reduced power for repeat analysis if one uses the assembled reference genome for the purpose of repeat finding.

**Fig 1:**
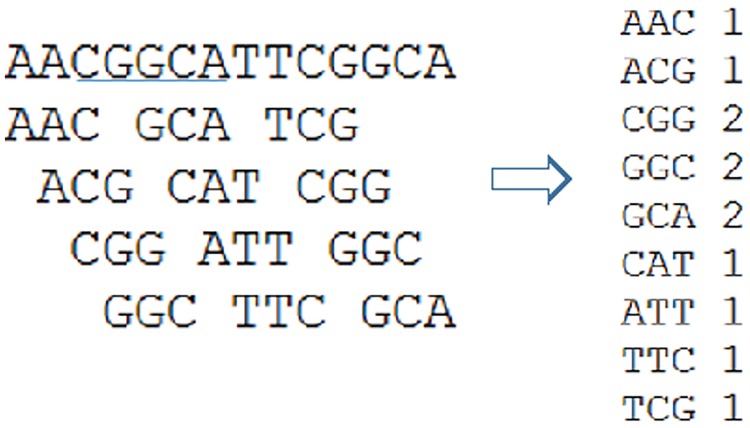
Illustration of the k-mer counting. Long sequence: a sequence read. Length-3 sequence: k-mer. Here, k = 3. The table on the right shows the k-mer counting result.

There are also methods which directly assemble repeats from sequence reads. RepARK [13] is such a method developed recently for repeat elements assembly. RepARK is based on k-mer counting. K-mers are substrings of *k* nucleotides. As shown in Fig 1, k-mer counting aims to count the occurrence of length-k substrings in all sequence reads. The result of k-mer counting is a vector *OCC* of size 4^k^, where *OCC*_*i*_ is the number of times the *i*-th k-mer appears in the reads. For example, in Fig 1, there is a single read. CGG appears two times while AAC and ACG appear once each. There exist efficient algorithms for k-mer counting, e.g. [15]. RepARK uses an approach which reconstructs segments of repetitive regions directly from sequence reads by first counting the k-mers from the sequence reads and then assembling all frequent k-mers (whose frequencies exceed some fixed threshold) [13]. The key idea is that k-mers in repeats may be more frequent than k-mers not in the repeats due to the high copy numbers of repeats. [13] showed that some contigs assembled this way are fairly long and many contigs can be mapped to the reference genome. Here, a contig is a segment of assembled genomes. This is encouraging since it demonstrates that estimation of repeats such as TEs can be done *de novo* from raw short-read sequencing data. However, as we will show in this paper, the method implemented in RepARK tends to only construct partial repeats. This may lead to considerable uncertainty when analyzing the evolution of repeat elements and to reduced detection rates of new repeat elements.

## Methods

**Fig 2:**
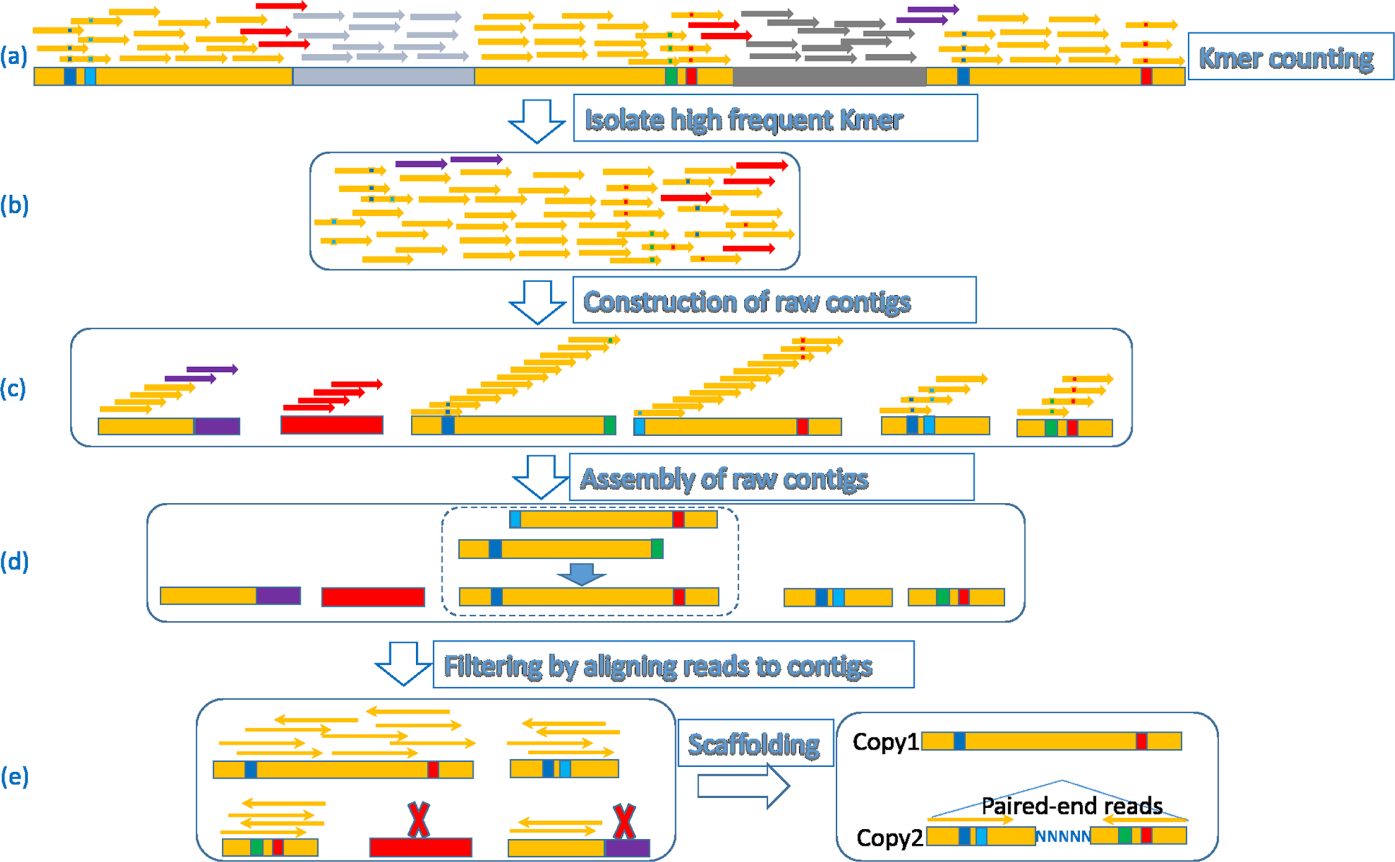
Illustration of the main steps of REPdenovo. Thick bars: genomic sequences. Thin bars: k-mers. K-mer counting step: yellow parts are repeats (with some mismatches). Colored squares within thick bars: mutations (substitutions and indels) within repeats.

The new repeat assembly method, REPdenovo, performs *de novo* estimation of low-divergent and highly frequent repeats from sequence reads. Similar to RepARK [13], REPdenovo first identifies the frequent k-mers and then assembles these k-mers. This step leads to a set of repeat contigs (called raw contigs). Raw contigs are the final results of RepARK. However, raw contigs are often only fragments of complete consensus repeats. This is because repeats usually contain regions of higher sequence divergence than other regions. K-mers within higher divergence regions tend to have much smaller frequencies and thus may not be identified to be frequent. The frequent k-mer assembly only leads to segments that have low divergence (i.e. more conserved) in repeat copies. Therefore, assembly of frequent k-mers alone does not produce contigs spanning complete repeats. To address this issue, REPdenovo performs a second assembly step by connecting raw contigs into long repeats. The key steps of REPdenovo algorithm are illustrated in Fig 2 and are explained below:

1. Assembly of raw contigs from frequent k-mers.
2. Merging of raw contigs into larger contigs ideally representing the entire repeat motif. This step is conceptually analogous to the idea of merging contigs into scaffolds in regular genome-assembly.
3. Verification and filtering of the assembled repeats. By aligning reads back to the constructed repeats and checking the read depth, some wrongly assembled repeats can be filtered out.

### 0.1 Construction of raw contigs

REPdenovo first constructs raw contigs directly from sequence reads by constructing a catalog of highly represented kmers, i.e. k-mers with frequencies over average k-mer frequency times a threshold value *f*_*K*_. The default value of *f*_*K*_ is 10, which means the frequency of a frequent k-mer is over 10 times the average k-mer frequency. This step could be improved in the future by using k-mer probabilities that take nucleotide or di-nucleotide frequencies into account. The current implementation uses Jellyfish [15] for k-mer counting, although other k-mer counting algorithms can also be used.

Once frequent k-mers are identified, the next step of REPdenovo is assembling frequent k-mers into contigs (called raw contigs). This is done by treating the frequent k-mers as sequence reads and then assembling these k-mers by existing short read assembly tools. Currently, Velvet [16] is used for this step. REPdenovo implements several additional techniques for more accurate construction of raw contigs and classification of repeats. First, REPdenovo takes a “frequency-based assembly” approach. That is, REPdenovo does not assemble all frequent k-mers in one step as in RepARK [13]. Instead, it groups and assembles k-mers with similar binned frequencies. A bin is a range of frequency based on a target frequency. By default, the range is [0.2f, 5f], where f is the target k-mer frequency. REPdenovo selects a number of evenly spaced target frequencies based on the collected k-mer frequencies from the reads as follows. REPdenovo starts from the k-mers in the highest frequency bin. Each time, REPdenovo assembles frequent k-mers within the current frequency bin. The range is then decreased (by default two times from the previous one) for the next round in a way that there is overlap in ranges of two consecutive steps. Users can also change the ranges in the settings of REPdenovo. Frequency-based assembly may reduce assembly error under the assumption that k-mers from the same repeat tends to have similar frequencies.

We use multiple k values (e.g. 20, 30 and 40) for assembly. Raw contigs assembled from different k-values are then combined to form a single list of raw contigs.

### 0.2 Assembly of raw contigs into long repeats

Most current next-generation sequencing platforms, like Illumina, generate paired-end reads with length around 100bp. Paired-end reads allow users to sequence both ends of a fragment and generate high-quality, alignable sequence data.

Empirical results show that most assembled raw contigs tend to be close to 100 bp in length for real sequence reads. However, many repeats are much longer than 100 bp. For example, many L1 elements in humans are 5 kbp or longer. Thus, raw contigs alone usually do not give complete repeat consensus sequences. To address this problem, REPdenovo performs a second assembly step by connecting the raw contigs into long repeats as follows.

For the raw contigs, we build a directed contig graph *G*, which is similar to the overlap graph in sequence assembly [17]. Each node in *G* corresponds to a raw contig. There is an edge from node *v*_1_ to *v*_2_ if there is significant overlap between contig *v*_1_ and contig *v*_2_. We say *v*_1_ has significant overlap with *v*_2_ if the length of the overlap between *v*_1_ and *v*_2_ is longer than a threshold value (15 bp by default) and the number of mismatches (substitutions and indels) is small (< 5% by default). The analysis is performed using standard pairwise sequence alignment based on dynamic programming, e.g. [18]. The overlap detection step allows errors in the overlapped regions of the raw contigs. This is because the overlapped regions of two connecting raw contigs usually don’t match exactly. Performing the alignment for all *O*(*n*^2^) pairs can be slow when *n* is large. REPdenovo therefore only aligns pairs of raw contigs containing common length *k*_0_ substrings. REPdenovo uses *k*_0_ = 5 in the current implementation. Such preprocessing speeds up the computation significantly in practice.

Overlap alone may not be very reliable especially when the length of the overlap region is small. To allow an edge from *v*_1_ to *v*_2_ in *G*, REPdenovo also requires the existence of read pairs where one end maps (using “bwa mem” with default parameters) to *v*_1_ and the other maps to *v*_2_, when such read pairs are expected given the insert size of the library and the relative positions of *v*_1_ and *v*_2_ in the merged contig. We use the default settings of BWA for reads mapping.

Once the contig graph *G* is constructed, REPdenovo then searches for long paths between two nodes in *G*. Each path corresponds to an assembled long repeat. There are often cycles in *G* and it is usually impractical to enumerate all paths in *G*. To address the issue of cycles, REPdenovo first finds the strongly connected components in *G*. A strongly connected component (SCC) contains one or multiple nodes where any two nodes are mutually reachable. Suppose we treat a SCC as a node. Then *G* implies a new graph *G*′, where the nodes of *G*′ are the SCCs and there is an edge from *SCC*_1_ to *SCC*_2_ if a node in *SCC*_2_ is reachable from a node in *SCC*_1_ in *G*. The definition of SCC ensures *G*′ is acyclic. We then run the standard topological sort algorithm (e.g. [19]) on each SCC (i.e. subgraph *G*_1_ containing only nodes in the *SCC*_1_). When *G*_1_ is acyclic, topological sort arranges the nodes of *G*_1_ in a linear topological order so that all edges of *G*_1_ point to the same direction in the linear order. That is, topological order means for every directed edge *u* → *v* from vertex u to vertex v in *G*, *u* is prior to *v* in the ordering. When *G*_1_ contains cycles, topological sort can still run but it does not lead to a perfect topological linear order. Our experience shows that while cycles exist in *G*, *G* is often near-acyclic and the strongly connected components are usually small. Thus, we simply rely on the linear order produced by the topological sort algorithm even when the linear order is not strictly topological order. REPdenovo then enumerates (possibly a subset of) paths that traverse one or several components in a heuristic way. The path finding generally follows the topological order when traversing the graph. Within each component, REPdenovo takes a heuristic approach for finding a valid path that allows the traversal to find long paths. In particular, when there are multiple edges to follow from the current node during the path finding, edges that agree with the linear order and lead to the nearest node in the linear order are preferred. To avoid cycles, REPdenovo assumes each raw contig may only appear in a path at most once. That is, each path contains distinct nodes in *G*. REPdenovo only outputs the maximal paths in *G* (i.e. paths that are not sub-paths of another path). Empirical results show that this path finding approach works reasonably well in practice.

### 0.3 Improving assembly quality by filtering

To further improve assembly REPdenovo uses two filtering steps. First, before assembling the raw contigs, contigs that have no (or very low) sequence read coverage are removed. Second, we truncate a raw contig if its coverage is uneven. We align the raw reads back to the raw contigs using “bwa mem” [20] with “-a” option. Then, we calculate the coverage for each base of each raw contig. If the average coverage is lower than a threshold value (2 by default), then this contig is considered to be wrongly assembled and is discarded. For example, in Part (e) of Fig 2, the raw contig marked in red color is discarded since it has no mapped reads. Sometimes a contig has uneven coverage, which means parts of the contig have high coverage and other parts have very low coverage (lower than the threshold). Such contigs are truncated so that only the high coverage parts are kept. For example, in Part (e) of Fig 2, the right part of the lower right raw contig (marked with the purple color) is truncated due to low read coverage.

### 0.4 Evaluation and comparison of methods

Throughout this paper, we use NCBI Blast (the output of blastn with default cutoff parameters which is considered to be “significant hits”) to compare a query sequence against a set of reference sequences. We define “matching cutoff” as the ratio between the length of the matched part (between the query sequence and the reference sequence) and the length of the query sequence. Notice that we use Blast searches in two different settings:

1. To compare a query sequence against a set of reference sequences such as the reference genome of a species or a set of estimated repeats (e.g. Blast a constructed repeat against Repbase). We call this use of Blast “Blast”.
2. Sometimes we want to search for a query sequence in the entire known nucleotide database at NCBI. We call this use of Blast as “NCBI Blastn”.

For a mapping between a repeat and the reference genome, we use a matching cutoff of 0.0 as default, which means we allow any matches that are considered to be significant by Blast (with default parameters). For other matchings, unless otherwise stated, the default matching cutoff is at least 85%. See the supplemental materials (S1 File) for more details about the settings.

In order to evaluate the performance of REPdenovo on repeat assembly, we first analyze raw sequence data from a human individual: individual NA12889 from the 1000 Genomes Project [21]. In the following, the repeats are assembled from the NA12889 reads, unless otherwise stated. To evaluate the consistency of REPdenovo, we also use REPdenovo to assemble repeats with three other 1000 Genomes individuals: HG01890, NA18641 and NA19206. See Table 1 for information on the reads data. See S1 File for the source of data. We first compare two different k-mer frequency cutoff *f*_*K*_ values of 10 and 100 to evaluate the performance of REPdenovo on different parameters. We thereafter mainly use *f*_*K*_ = 10 unless otherwise stated.

**Table 1:** Sequence reads information from four human individuals from the 1000 Genomes Project. # of reads: in millions. Coverage: average sequence depth per base.

Individual	Population	# of reads	Read length	Coverage
**NA12889**	CEU	229M	101	7.2
HG01890	ACB	394M	100	12.3
NA18641	CHB	254M	101	8.0
NA19206	YRI	172M	100	5.4

## Results

The results section is organized as follows: First, we evaluate the performance of REPdenovo using sequence reads from four human individuals. We show that the found repeats are likely to be real and many are absent from the human reference genome. Then, by comparing with repeat annotations stored in existing repeat libraries and latest long human sequence reads, we identify and validate a set of potentially novel repeats in the human genome that are not included in existing repeat annotations. At last, we show REPdenovo outperforms RepARK in terms of the accuracy and completeness of the constructed repeats.

### 0.5 Constructed human repeats are likely to be real

#### Classification of constructed repeats

We use REPdenovo to construct consensus repeats from reads data of the human individual NA12889. For each repeat, we address the following two questions:

1. Is the repeat mappable to the human reference genome?
2. Can the repeat find homologs in a NCBI Blastn search?

In general, if a repeat can be mapped to the human reference, the repeat is more likely to be real (i.e. not introduced by assembly artifact). Since many existing methods rely on the reference genome in repeat analyses, reads identified from reference genomes are likely to be incorporated in such analyses (e.g., in Repbase). Novel repeats (i.e, not previously reported in humans) in contrast are not mappable to the human reference. However, inferred new repeats that do not map to the genome may in reality be assembly artifacts. In order to identify repeats that are more likely to be real, we search for homologs of the each consensus repeat using NCBI Blastn. If the consensus repeat is represented in the *nt* NCBI database it is unlikely to be an assembly artifact.

For NA12889, 5,479 out of 6,200 for *f*_*k*_ = 10, and 589 out of 669 for *f*_*k*_ = 100, estimated repeats from REPdenovo are mappable to the human genome. Furthermore, 190 out of 721 for *f*_*k*_ = 10, and 78 out of 80 for *f*_*k*_ = 100 can find high quality NCBI Blastn hits even when they are not mappable to the human reference (see Table 2 for details). This suggests that the vast majority of constructed repeats are real and not assembly artifacts. It also suggests the possibility that a significant number of human repeats may be absent from the current human genome reference.

**Table 2:** The number of classified repeats constructed by REPdenovo on four different human individuals for *f*_*K*_ = 10 and 100. Classified into: (i) mappable to the reference genome, (ii) unmappable to the reference but have NCBI Blastn hits, and (iii) unmappable to the reference and no NCBI Blastn hits. The repeats in (ii) and (iii) may potentially be previously unknown repeats.

*f*_*K*_	Individuals	Mappable	Unmappable		Total
			NCBI Blastn hits	No NCBI Blastn hits	
10	**NA12889**	5,479	**190**	**531**	6,200
	NA18641	5,626	189	764	6,579
	NA19206	6,055	150	603	6,808
	HG01890	5,606	171	691	6,468
100	NA12889	589	78	2	669
	NA18641	610	83	8	701
	NA19206	646	57	11	714
	HG01890	609	80	6	695

#### Length distribution of matched repeats

**Fig 3:**
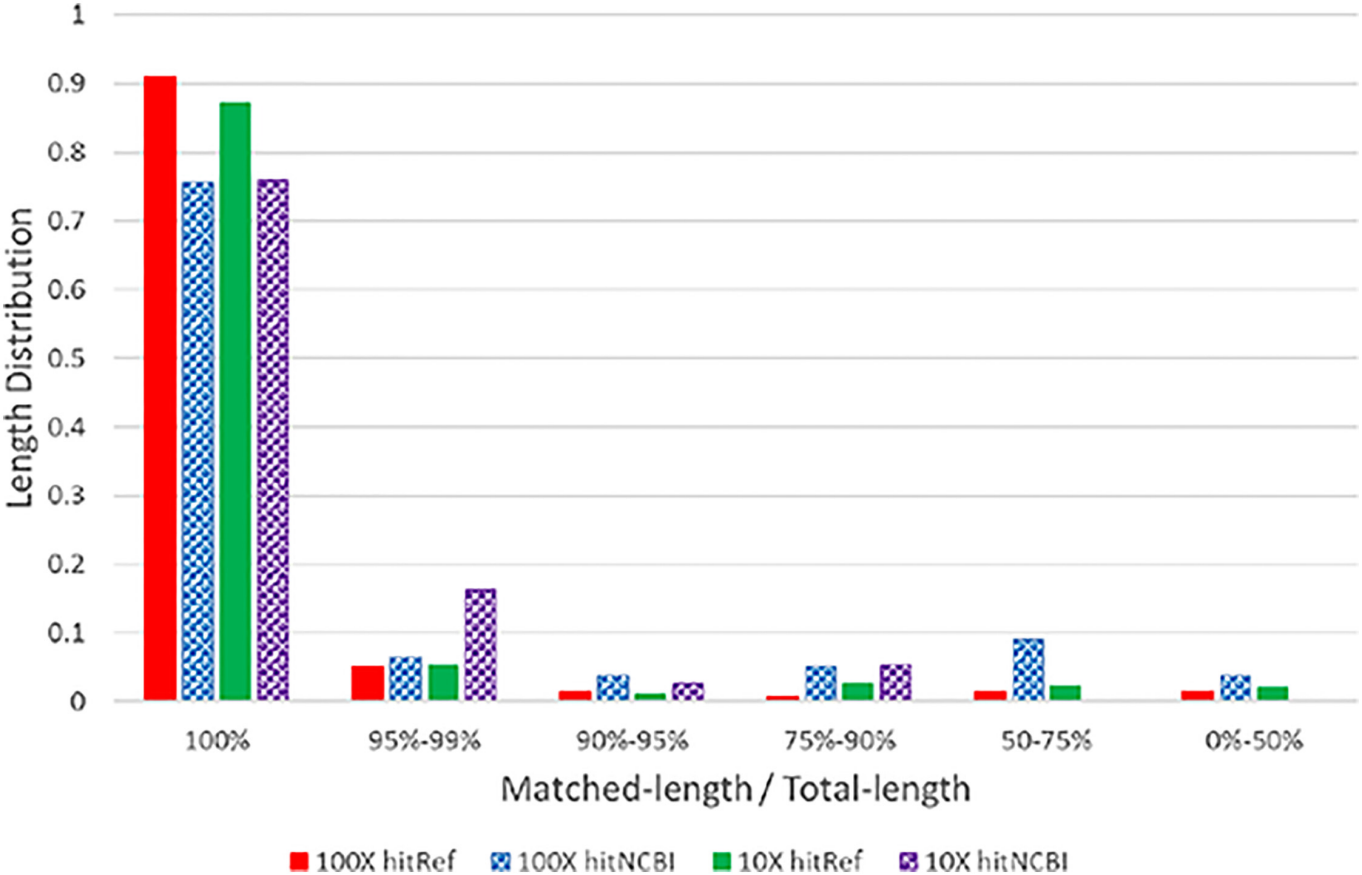
Distribution of repeat matching lengths relative to their total length for f_K_ = 10 and f_K_ = 100. Solid bars: repeats mappable to the reference genome. Bars with patterns: repeats unmapped to reference and having NCBI Blastn hits. The figure shows the relative matching length as the mapping ratio (0%-100%), which is the ratio between the length of mapped part and total length of the repeat. A majority of constructed repeats can match fully to the reference genome or have NCBI Blastn hits.

Fig 3 shows the length distribution of the human repeats that are either mappable to the reference or have good NCBI Blastn hits for *f*_*K*_ = 10 and 100. The solid bars show the matching length distribution for repeats that are mappable to the reference. The bars with patterns are for those not mappable to the reference but having good NCBI Blastn hits. Most repeats have matching ratios of 100% or nearly 100% (i.e. the entire repeat can be mapped) for both reference matching and NCBI Blastn hits. That is, when a repeat is matched, it is quite likely the whole repeat can be matched to the reference genome or to the NCBI *nt* database. This suggests assembly accuracy of the constructed repeats may be high.

#### REPdenovo constructs repeats with high copy numbers and low sequence divergence

**Fig 4:**
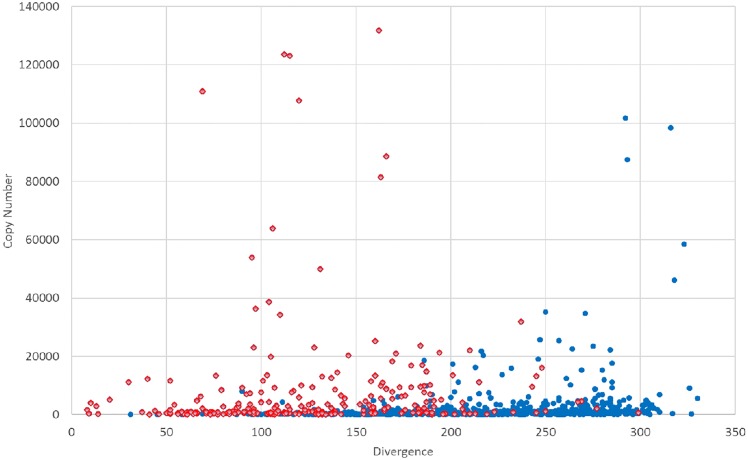
Hits of Repbase repeats found by REPdenovo. X axis: divergence rate (mismatches per 1,000 bases) of repeats given by Repbase. Y axis: number of copies from the UCSC genome browser annotation. Dots: Repbase repeats. Red dots: hits found by REPdenovo. Blue dots: repeats not found by REPdenovo.

REPdenovo works better for low divergent than for high divergent repeats. To illustrate this, we show a distribution of constructed repeats as a function of copy number and repeat divergence in Fig 4. We use matching cutoff 0.0 when comparing with Repbase repeats. To get the copy number and divergence of repeats, we use UCSC annotation [22], which utilizes a copy number generated by RepeatMasker. There are 1,119 human repeats in Repbase. Here, we only use 1,001 repeats out of all repeats which exist in the UCSC annotation. 283 out of the 1,001 repeats have a hit among the constructed repeats. From Fig 4, it is clear that most of the hits are on repeats of low divergence and high copy number.

#### Consistency of different human individuals

To evaluate the consistency of REPdenovo, we use REPdenovo to construct repeats from four human individuals using data from the 1000 Genomes Project. The results are shown in Table 2. It can be seen that the numbers of repeats in different categories are overall consistent for different individuals for both *f*_*K*_ = 10 and *f*_*K*_ = 100 cases. Differences might reflect the variations in repeats in different human individuals, differences in read quality and sequencing depth between individuals.

### 0.6 Comparison to Repbase and RepeatMasker and finding novel repeats in human genome

One of our main goals is finding novel human repeats that are previously unknown. For this purpose, we first map the constructed repeats to human reference. As expected, repeats that are mapped to the reference are more likely to find matches in Repbase. Repeats unmappable to human reference may be novel. We rely on two means to validate whether a repeat is novel: (i) perform NCBI Blastn search: a repeat with good hits is likely to be real; (ii) compare with the latest long human reads: if a repeat matches the long reads data, it is likely to be real.

#### Repeats with Repbase hits and/or masked by RepeatMasker

Table 3 shows the overall results on the number of constructed repeats. For both mappable and unmappable repeats, first we examine whether the repeats can find matches in Repbase. Then we run RepeatMasker on the inferred repeats to examine whether the repeats can be classified into a particular type. Here, the matching cutoff for NCBI Blastn is 0.0, and is also 0.0 for both Repbase hits and “masked” by RepeatMasker. As expected, repeats mappable to the reference are more likely to find matching Repbase repeats, while unmappable repeats have no Repbase hits. RepeatMasker can give results on many repeats, although sometimes only small parts of repeats can be masked.

**Table 3:** Numbers of repeats that hit Repbase (with matching cutoff 0.0) and masked by RepeatMasker (with matching cutoff 0.0). We classify the repeats based on whether they are mappable to the human reference and whether they have matches in Repbase. Masked: RepeatMasker can classify the repeat. Unmasked: RepeatMasker cannot classify the repeat.

*f*_*K*_	Mappable to reference				Unmappable to reference			
	Hit Repbase		No-hit Repbase		Hit Repbase		No-hit Repbase	
	Masked	UnMasked	Masked	UnMasked	Masked	Unmasked	Masked	Unmasked
10	3,426	26	1,239	788	0	0	683	38

#### Potentially novel repeats in human

There are 190 repeats for NA12889 in Table 2 that do not have significant hits on the human reference (GRCh37) but find significant and nearly complete hits in NCBI Blastn. In Table 4 (the first column) we give out a detailed statistic of these hits. Note that, one repeat may have more than one hits and here only the top one (blast with option “-max_target_seqs 1”) is used. We believe that these 190 repeats are potentially novel repeats in human, although some may be assembly artifacts. We run Blast on these 190 repeats against the constructed repeats of the other three individuals. Out of the 190 repeats, 152, 156 and 157 repeats can find Blast hits on NA18641, NA19206 and HG01890, respectively. We also run RepeatMasker on these 190 repeats. Among the 190 repeats, 129 are masked, and among these, 101 are identified to be “Satellite repeat”, 25 are “Simple repeat”, and three are “centromeric”. We note that RepeatMasker tends to mask the shorter ones among these 190 repeats. This is illustrated in Table 5. Note that fewer repeats are masked by RepeatMasker than those in Table 3 because here we require near complete matching when running RepeatMasker.

**Table 4:** Classification of the 190 un-mappable repeats with NCBI Blastn hits. The numbers in parentheses are the numbers of repeats in each category.

NCBI Blastn hit description	All (190)	Long read reference hits (164)	Masked (129)
Epstein-Barr virus	*2*	*0*	*0*
Homo FOSMID clone	8	8	4
Homo BAC clone	8	8	2
Homo related	35	33	29
Gossypium hirsutum clone	2	2	0
Haemonchus placei genome	51	51	50
Human herpesvirus	*19*	*0*	*0*
Onchocerca flexuosa genome	6	5	3
Protopolystoma xenopodis genome	18	18	5
Spirometra erinaceieuropaei genome	40	38	36
Toxoplasma gondii ME49	1	1	0

**Table 5:** Length distribution of the 190 potentially novel repeats. The numbers in parentheses are the numbers of repeats in each category. For each range of repeat lengths, the number is the percentage of repeats falling in the range.

length	All (190)	Mappable to long reads reference		Masked by RepeatMasker	
		Yes (164)	No (26)	Yes (129)	No (61)
100–300	79.4	87.8	27.0	86.8	63.9
301–500	9.5	9.2	11.6	9.3	9.9
501–750	1.1	0.6	3.8	0.8	1.6
751–1,000	2.6	2.4	3.8	3.1	1.6
1,001–1,500	0.5	0	3.8	0	1.6
1,501–2,000	1.1	0	7.7	0	3.3
2,001–5,000	1.1	0	7.7	0	3.3
5,001–50,000	4.7	0	34.6	0	14.8

#### Compare novel repeats with long reads data

To further validate that the 190 repeats indeed are present in the human genome, we compare them against the Pacific-Bio long reads released in [23]. These reads are generated from a human hydatidiform mole cell line (CHM1) from SMRT sequencing [23]. As the error rate of the Pacific-Bio reads is usually high, we use the released error-corrected reads which are corrected by a multiple read alignment procedure. 164 and 161 out of the 190 repeats match one or more of the Pacific-Bio reads with matching cutoff 0.85 and 0.95, respectively.

There are two recently released human reference genomes based on Pacific-Bio reads, and we also Blast the 190 novel repeats against these. One reference is directly assembled from Pacific-Bio reads [23], while the other is based on extending the gaps in GRCh37 using Pacific-Bio reads [24]. We get different results when blasting the repeats against these two references. For the directly assembled reference [23], we find 164 hits with matching cutoff 0.85, while for the patched reference [24] we only find 1 hit using the same cutoff. It is possible that the reference constructed by GRCh37 has missed some hard-to-assemble regions.

There are 531 repeats in Table 2 for NA12889 that cannot map to the reference and have no NCBI Blastn hits. We also Blast these 531 repeats against the corrected long reads. 18 and 16 out of the 531 repeats match at least one long read with matching cutoff 85% and 95%, respectively. Thus, there appears indeed to be a small number of novel valid repeats even among the ones that do not have a significant NCBI Blastn hit.

Overall, the fact that a majority of repeats match at least one Pacific-Bio read with at least 95% identity across the entire repeat sequence provides additional support for our belief that the majority of inferred reads are real and are not assembly artifacts. However, we note that this does not completely rule out the possibility of assembly errors.

#### Further analysis of potentially novel repeats in Human

Among the 26 repeats with no long reads hits, but with significant NCBI Blastn hits, the average NCBI Blastn identity is 99.5%. Average coverage (alignment length/repeat length) is 98.5%. The largest E-value of the 26 repeats is 2.0e-49 and 17 repeats have E-value reported to be 0.0. We also note that the average length of the 26 repeats is 7,988 bp, while the average length of all 190 repeats is 1,266 bp (see Table 5). This may suggest that the long repeats are poorly assembled even in the Pacific-Bio reference genome.

We classify all 190 potentially new repeats according to whether they find matches in the new reference sequence or are masked by RepeatMasker in Table 4. The general pattern mirrors that for the 26 repeats with not long read hits. However, we now observe an increased proportion of repeats that previously have been classified as human, for example to sequenced BAC clones that are not incorporated into an assembly. We also observe an increase in hits to specific blood parasites, particularly *Haemonchus placei* and *Spirometra erinaceieuropaei*.

We see that a substantial proportion of the hits are viral. The EBV virus is not surprising as it has been used to transfect the sequenced cell lines. Matches to other viruses (e.g., herpes virus) may be caused by contamination of the sequencing libraries or the cell cultures or by homology with the transfecting virus. We note that three herpesvirus have high sequence homology with parts of two EBV virus. We also note that there is no hits to the long reads reference for the repeats among the viruses. This supports our hypothesis that these repeats in fact reflect homolgy with transfecting viruses or contamination.

The remaining hits are all blood parasites. It is likely that these are real repeats that have been incorporated into the parasite assemblies by error, as sequencing of parasites typically is based on samples contaminated with the host DNA, which can be removed by filtering. However, if the host reference sequence is missing the sequence motif, such filtering may fail. Repeats motif not present in the reference genome of the host and not caught by RepeatMasker are likely to fall into this category.

We take a closer look at the 26 repeats that do not hit the new long reads reference. Table 6 shows the top hits from NCBI Blastn for these 26 repeats, and the number of repeats masked (all are masked as “Simple repeats”) by RepeatMasker for each type. The numbers inside parentheses are the number of occurrences of the repeats of the specific types.

**Table 6:** Classification of the 26 (out of the 190 potentially novel repeats) repeats that have no Blast hits on long reads reference. The numbers in parentheses are the numbers of repeats in each category.

NCBI Blastn hit description	All (26)	Masked (18)
Homo sapiens isolate satellite	2	2
Human herpesvirus	19	11
Onchocerca flexuosa genome	1	1
Spirometra erinaceieuropaei genome	2	2
Epstein-Barr virus	2	2

### 0.7 Comparison of assembly quality of REPdenovo and RepARK

We compare REPdenovo to RepARK repeat assemblies by comparing both to the repeats represented in Repbase [11]. In the following, “hits” refer to constructed repeats that are represented in Repbase, and we use the following metrics to compare REPdenovo to RepARK:

1. The number of Repbase hits with > 85% sequence identity across the length of the Repbase represented repeat sequence.
2. Average Repbase coverage. For a Repbase hit, this is the average fraction of the Repbase repeat covered by the assembled sequence. For a single position of a hit, there can be multiple assembled repeats covering it. When calculating the average coverage, we use the set of non-overlapping assembled repeats that achieve the largest coverage. This statistic can be computed by a simple greedy algorithm.
3. Average Repbase coverage by the longest assembled repeat. One repeat in Repbase may be covered by several constructed repeats. When calculating the average coverage, we choose the longest one. This statistic is used to examine how well the methods can construct long repeats (not just fragments of repeats).
4. N50 of the assembled repeats.

**Fig 5:**
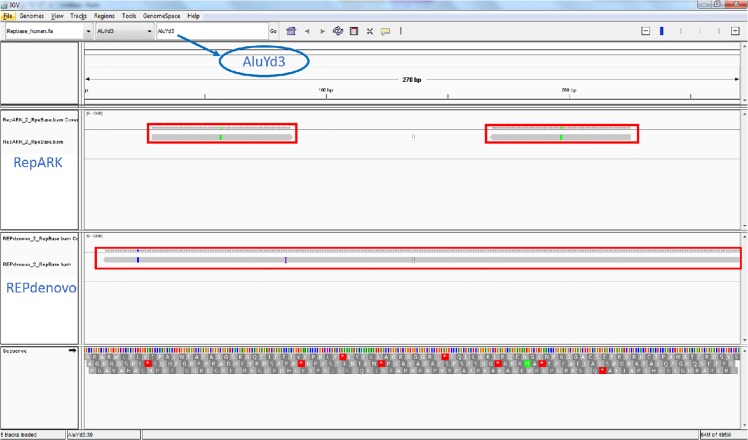
Assembled repeats matching AluYd3 (a Repbase repeat) by REPdenovo (bottom panel) and RepARK (top panel). The matched assembled repeats are shown on their mapped positions where the AluYd3 consensus repeat sequence serves as the reference.

We run REPdenovo and RepARK with same kmer frequency and assembly parameters (both use velvet [16] as the assembler). The results of REPdenovo and RepARK on individual NA12889 are given in Table 7. REPdenovo outperforms RepARK in all quality metrics. In particular, REPdenovo can assemble longer repeats while RepARK tends to assemble smaller fragments of repeats. REPdenovo also achieves higher average coverage of Repbase repeats than RepARK. The N50 of REPdenovo assembled repeats is about 27 times of that of RepARK assembled repeats. As an example, we use one Repbase repeat, AluYd3, for an illustration. This is shown in Fig 5, generated by mapping the assembled repeats on Repbase repeats and then visualizing with the program IGV [25]. The length of the AluYd3 repeat is approximately 270 bp. In this case REPdenovo almost assembles the complete repeat while RepARK only assembles two small fragments. This illustrates the stark difference in completeness and length of constructed repeats between the two methods as measured by N50 and other statistics.

**Table 7:** Assembly quality comparison of REPdenovo and RepARK. *N*: the number of assembled contigs. *N*_*h*_: the number of complete Repbase hits from the *N* repeats (with 85% coverage cutoff). $\bar{C}$: average coverage of hits. *C*_*m*_: maximum coverage of hits by single assembled repeats. N50: N50 of assembled repeats.

*f*_*K*_	Method	*N*	*N*_*h*_	$\bar{C}$	*C*_*m*_	N50
10	REPdenovo	6,200	**91**	0.88	0.53	**3,141**
	RepARK	7,894	**1**	0.74	0.06	**116**

To further compare the performance of REPdenovo and RepARK, we run REPdenovo and RepARK on four 1000 Genomes individuals: NA12889, HG01890, NA18641 and NA19206. We use Blast to identify matches in Repbase and investigate how well long repeats are constructed with matching cutoff *t*_*L*_.

Table 8 shows the repeat assembly performance on all four individuals (including NA12889). We can see that results from REPdenovo overall keep consistent as the matching cutoff *t*_*L*_ changes from 0.0 to close to 1.0 (by default, *t*_*L*_ is chosen to be 0.85). Also, REPdenovo outperforms RepARK in terms of the completeness and the number of long repeats constructed. This is benefit from the assembling raw contigs and filtering steps. As copies of a repeat are diverged from each other, lots of short pieces (of copies) will be assembled because of the variations on the copies, and assembling these raw contigs will help to construct the complete repeats, while RepARK only reports these pieces. Thus, REPdenovo works better for constructing more diverged repeats. However, it is still possible for REPdenovo to wrongly assemble contigs, even though there is a filtering step.

**Table 8:** The number of repeats in Repbase that match (over the minimum threshold *t*_*L*_) one *de novo* repeat. The numbers outside and side the parentheses are REPdenovo and RepARK results, respectively. *t*_*L*_: matching cutoff.

*f*_*K*_	*t*_*L*_	NA12889	HG01890	NA18641	NA19206
10	0.95	86 (1)	81 (0)	84 (0)	84 (1)
10	0.85	91 (1)	87 (0)	94 (0)	94 (1)
10	0.75	102 (1)	100 (1)	104 (1)	102 (3)
10	0.5	141 (9)	138 (10)	135 (12)	139 (12)
10	0.0	295 (278)	307 (294)	312 (293)	313 (296)

### 0.8 Running time

For a small genome and low-coverage sequence data, REPdenovo usually runs fast. It takes about 19 hours (including the k-mer counting time) to analyze the human individual NA12889 (read length 100bp with read depth 7.2X) on a 3.2 GHz eight core Xeon X5482 computer with 32G of memory.

## Discussions and Conclusion

In this paper, we develop REPdenovo, a new repeat analysis approach that reconstructs repeat sequences from raw sequence reads and does not rely on prior knowledge of repeats. REPdenovo can be applied to genomes that have been sequenced but for which no good reference genomes and repeat annotations are available. REPdenovo improves upon a previous approach, RepARK, by providing better assemblies of repeat consensus sequences in terms of completeness and number of long repeats constructed, as demonstrated by our analyses of human annotated repeats. REPdenovo can assemble full (or nearly full) repeat consensus repeats, while RepARK usually only produces small fragments of long repeats (see Tables 7 and 8). This is especially important for downstream analyses of the identified repeat sequences. While REPdenovo may only identify recently expanded repeat families, these are also the families that are of greatest interest in comparative studies, as older repeats tend to be shared among species.

Like most bioinformatics tools, REPdenovo requires specification of several parameters, which can significantly affect the results. The most important parameter is the relative k-mer frequency cutoff *f*_*K*_, which specifies the lowest k-mer frequency that a k-mer is considered to be frequent and assembled. The default value of *f*_*K*_ is 10, which means the frequency of a frequent k-mer is over 10 times of the read depth. When a higher value is used for *f*_*K*_, fewer repeats will be assembled. Also, the running time of REPdenovo will increase when *f*_*K*_ decreases. Another important parameter is *L*_*K*_, the length of k-mers. There is no rigorous way for choosing a single value of *L*_*K*_. Shorter k-mers are more robust against variations within repeats but may give less accurate assemblies due to ambiguity in assembly process. Longer k-mers may give more accurate assemblies but may miss some segments that contain more variations within the repeats. Thus, REPdenovo uses multiple *L*_*K*_ values when assembling raw contigs, while RepARK only uses a fixed *L*_*K*_ value (i.e. 30). Table 9 shows the number of repeats in Repbase that match (over the minimum threshold 0.85) one *de novo* repeat for different *L*_*K*_. The results show that different *L*_*K*_ values may generate different sets of repeats, and combining these repeats may provide more accurately assembled repeats.

**Table 9:** The number of repeats in Repbase that match (over the minimum threshold 0.85) one *de novo* repeat for different k-mer length *L*_*K*_. By default, REPdenovo use different k-mer length (29, 39, and 49) together, and its result is marked as “Combined”.

*L*_*K*_	21	29	39	49	59	69	79	89	99	Combined
Hit Repbase	13	49	61	71	75	71	54	57	46	91

We applied the method to human data and identified 190 potentially new repeats. We note that top Blast hits are non-human for some REPdenovo assembled repeats. For example, the top two hits for one assembled human repeat from NA12889 are for Onchocerca Flexuosa (a deer parasite) and Protopolystoma Xenopodis (an amphibian parasite). We also find the assembled repeats that have top Blast hits (with 100% coverage) on Onchocerca Flexuosa and Protopolystoma Xenopodis exist in the other three human individuals as well. One explanation is that there are homologous repeats with high sequence identity between humans and the parasites, perhaps because these are sequences that have jumped genomically, through unknown mechanisms, between hosts and parasites. A more likely explanation is that these are repeats caused by human contamination in the parasite sequencing projects.

Moreover, the newly available long Pacific-Bio reads provided additional support that the novel human repeats we constructed may indeed be real. For the 190 potentially novel human repeats, 129 repeats are masked by RepeatMasker to be mostly simple repeats or satellite repeats. Further studies are needed to find the types of the remaining repeats.

## Supporting Information

S1 File This file provides the supplementary information. (PDF)
