# Supplementary material for: REPdenovo: Inferring De Novo Repeat Motifs from Short Sequence Reads
Source: PLoS One. 2016 Mar 15;11(3):e0150719. doi: 10.1371/journal.pone.0150719 (PMC4792456; doi:10.1371/journal.pone.0150719)
Supplement: S1 File — (PDF) [file pone.0150719.s001.pdf]

REPdenovo: Inferring *de novo* repeat motifs from short sequence reads

Chong Chu [1], Rasmus Nielsen [2,*] and Yufeng Wu [1*]
[1] Department of Computer Science and Engineering, University of Connecticut, Storrs, CT 06269, U.S.A.
[2] Department of Integrative Biology, University of California, Berkeley, Berkeley, CA 94720, USA.

1. Data used in the experiments

1.1 Sequencing data

1.1.1 Sequenced short reads

For short reads, four human individuals: NA12889, NA19206, NA18641, and HG01890 are used in the experiments. All these four individuals are downloaded from The 1000 Genomes Project. See Table S1.

Table S1. Download URL for the four individuals used in the experiments

Individual	URL for Downloading
NA12889	ftp://ftp.1000genomes.ebi.ac.uk /vol1/ftp/phase3/data/NA12889/sequence_read
NA18641	ftp://ftp.1000genomes.ebi.ac.uk /vol1/ftp/phase3/data/NA18641/sequence_read
NA19206	ftp://ftp.1000genomes.ebi.ac.uk /vol1/ftp/phase3/data/NA19206/sequence_read
HG01890	ftp://ftp.1000genomes.ebi.ac.uk /vol1/ftp/phase3/data/HG01890/sequence_read

1.1.2 Pacbio long reads

For long reads, we download the corrected Pacbio long reads from: http://gembox.cbcb.umd.edu/mhap/data/human.polished.fastq.bz2

1.2 Reference genome

1.2.1 Reference genome assembled from short reads

We use GRCh37 as the default reference genome, which is download from The 1000 Genomes Project: ftp://ftp.1000genomes.ebi.ac.uk/vol1/ftp/technical/reference.

1.2.2 Reference genome assembled from Pacbio long reads

We also two reference genomes assembled from Pacbio long reads to validate the novel repeats constructed by REPdenovo. The first one is directly assembled from corrected Pacbio long reads, which is downloaded from:
http://gembox.cbcb.umd.edu/mhap/asm/human.quiver.all.fasta.gz
The other one is patched from GRCh37, which is downloaded from:
http://eichlerlab.gs.washington.edu/publications/chm1-structural-variation/data/GRCh37_patched/GRCh37_patched.fasta

1.3 Repeats library

1.3.1 Repbase
We use the latest released version (RepBase19.08.fasta) which is downloaded from:
http://www.girinst.org/repbase/

1.3.2 UCSC annotation
In Figure 4 of the main paper, we show the divergent rate of the repeats in Repbase. To get the divergence of each repeat, we use the released UCSC annotation, which contain the divergence (represented by the mismatch per thousand bases) of each copy, and the median one is used as the divergence of this repeat. The released UCSC annotation is downloaded from:
http://hgdownload.soe.ucsc.edu/goldenPath/hg38/database/rmsk.txt.gz

2. Commands used in the experiments

2.1 Run NCBI Blast

The latest version of "blastn" (ncbi-blast-2.2.30+) is used in the experiments. When running we use the default settings for Blast. Blast will reports all the significant alignments. But for different experiments, we may want to filter out some reported alignments. So we add a "matching cutoff" to filter some unnecessary hits. We define "matching cutoff" as the ratio between the length of the matched part (between the query sequence and the reference sequence) and the length of the query sequence.

2.1.1 Blast between a query and a subject
When to check whether a sequence can be aligned to another sequence or reference genome, we use command:

“ncbi-blast-2.2.30+/bin/blastn -query $1 –subject $2 -outfmt 6 -max_target_seqs 1”, where $1 represents the query sequence and $2 represents the subject sequence.

2.1.2 Blast a query against the “nt” library

To validate a repeats is novel or not, we first align the the repeats to the reference genome. For those unmapped ones, we blast them against the NCBI “nt” library to check whether we can find some hits. To do this we use command:
“ncbi-blast-2.2.30+/bin/blastn -query $1 -db nt -outfmt 6 -max_target_seqs 1 -out $2 -num_threads 5”, where $1 represents the query sequence and $2 is the output file.

2.2 Run RepeatMasker

We use the latest version of RepeatMasker (RepeatMasker-open-4-0-5.tar.gz) to mask the constructed repeats. To run, we use command:
“RepeatMasker -species $1 $2”, where $1 is the species name, here we use “human”; $2 is the repeats file in fasta format.

2.3 Run REPdenovo

See the released REPdenovo manual at:https://github.com/Reedwarbler/REPdenovo

2.4 Run RepARK

To run RepARK, we use command:
“perl RepARK-master/RepARK.pl -p $1 -t $2 -n -d -o $3 -l $4 -l $5”, where $1 is the number of threads to run Jellyfish; $2 is the kmer frequency threshold; $3 is the output file; $4 and $5 is the raw reads file for paired-end reads. Note that, by default RepARK will automatically calculate a kmer frequency threshold, but when we try in our data, RepARK failed to run. In the comparison, we use 10 and 100 for $2, which are the same settings as REPdenovo.

2.5 Run BWA and samtools

Version 0.7.12 of bwa is used to align the reads back to the constructed repeats. “mem” with default option is used.

“samtools” version 1.2 is used in all the analysis.
